# Supplementary material for: Iron Status of Burkinabé Adolescent Girls Predicts Malaria Risk in the Following Rainy Season
Source: Nutrients. 2020 May 16;12(5):1446. doi: 10.3390/nu12051446 (PMC7284973; doi:10.3390/nu12051446)
Supplement: Supplementary file 1 [file nutrients-12-01446-s001.pdf]

SUPPLEMENTARY FILE 1

ADDITONAL ANALYSES

Tables showing regression slopes for:

Figure 3 (Supplementary Table S1) Menarche effects for iron biomarkers

Figure 4 (Supplementary Table S2) Menarche effects for RDT and malaria parasitaemia

Figure 5 (Supplementary Table S3) Menarche effects (menarcheal - non menarcheal) for parasite prevalence by standardised iron biomarker levels

CONSORT Checklist

Supplementary Table S1: Regression slopes and menarche effects (menarcheal - non menarcheal) for iron biomarkers (Figure 3).

Iron Biomarker	Slope (per year) (95%CI)	Menarche effect (95%CI)
log(adjusted ferritin) (%)	-6.0 (-8.8 - -3.2)	-6.9 (-21.9 - 8.1)
log(sTfR/log ferritin) (%)	2.4 (0.7 - 4.0)	-0.4 (- 9.1 - 8.44)
log(hepcidin) (%)	-8.3 (-12.2 - -4.3)	-20.9 (-41.7 - -0.1)
Adjusted body iron stores (mg/kg)	-0.22 (-0.34 - -0.10)	-0.23 (-0.87 - 0.41)

Log-transformed variable slopes are expressed as %/year.

Supplementary Table S2: Regression slopes and menarche effects (menarcheal - non menarcheal) for RDT and malaria parasitaemia (Figure 4).

Parameter	Slope (95%CI)	Menarche effect (95%CI)
RDT by age	0.90 (0.84 - 0.96)	1.27 (0.89 - 1.83)
Parasitaemia by age	0.88 (0.82 - 0.95)	0.79 (0.56 - 1.14)
RDT by BMI Z-score	0.88 (0.74 - 1.05)	1.33 (0.90 - 1.96)
Parasitaemia by BMI Z-score	0.95 (0.80 - 1.13)	0.77 (0.52 - 1.13)

Slopes are expressed as odds ratios per year or BMI Z-score (per SD)

Supplementary Table S3: Regression slopes and menarche effects (menarcheal - non menarcheal) for parasite prevalence by standardised iron biomarker levels (Figure 5).

Iron Biomarker	Slope (per SD) (95%CI)	Menarche effect (95%CI)
log(ferritin)	1.53 (1.11 - 2.11)	0.68 (0.48 - 0.97)
log(sTfR/log ferritin)	0.42 (0.23 - 0.76)	0.67 (0.48 - 0.95)
log(hepcidin)	1.23 (0.98 - 1.55)	0.68 (0.48 - 0.97)
Body iron (mg/kg)	1.05 (1.02 - 1.09)	0.68 (0.48 - 0.97)

Slopes are expressed as odds ratios per standard deviation of sample distribution.

CONSORT CHECKLIST

PAPER SECTION<br>Topic	Item	Description	Reported Page(s) /Table or Figure
TITLE<br><br>ABSTRACT	1a<br><br>1b	Iron status of Burkinabé adolescent girls predicts malaria risk in the following rainy season<br>Unstructured	1<br><br>1
INTRODUCTION	2a<br><br>2b	Scientific background and explanation of rationale<br>Specific objectives	2<br><br>2
METHODS<br>Design	 3a<br>3b	 Trial design<br>Changes to trial design	 3; Ref 7<br>N/A
Participants	4a<br>4b	Eligibility<br>Study setting	3<br>3
Interventions	5	Description	3
Outcomes	6a<br>6b	Pre-specified<br>Changes to trial outcomes	4<br>5
Sample Size	7a<br>7b	Sample size determination<br>Interim analyses	4<br>Results 6-12
Randomisation	8a<br>8b	Sequence generation<br>Type	Ref 7
	9	Allocation concealment mechanism	Ref 7
	10	Implementation	Ref 7
Blinding	11a<br>11b	Who and how blinded<br>Similarity of the interventions	Ref 7<br>Ref 7
Statistical methods	12a<br>12b	Main outcomes<br>Sub-group analyses	5<br>Tables 1-4<br>Figures 2-5<br>Suppl tables S1-S3
RESULTS<br>Participants	 13a<br>13b	 Participant flow diagram<br>Losses and exclusions	 7<br>7
Recruitment	14a<br>14b	Periods of recruitment<br>Reasons for stopping	2,3<br>3.4
Participant characteristics	15	Baseline data table	6
Denominators	16	Numbers analysed	6, Table1
Outcomes	17a<br>17b	Effect sizes and estimations<br>Binary outcomes	Tables 2-3; Figures 2-5<br>Table 1; page 5
	18	Ancillary analyses	Tables S2-S4
Harms	19	Adverse events	Ref 13
DISCUSSION<br>Limitations	 20	 Eg Imprecision, bias	 12,13
Generalisability	21	Internal/external validity	12,13
Interpretation	22	Consistency with other studies	12
OTHER	23	Trial registration	3
	24	Availability of protocol	On request
	25	Funding/role of funder	14
